# Virtual patient‐specific QA with DVH‐based metrics

**DOI:** 10.1002/acm2.13639

**Published:** 2022-05-15

**Authors:** Lam M. Lay, Kai‐Cheng Chuang, Yuyao Wu, William Giles, Justus Adamson

**Affiliations:** ^1^ Medical Physics Graduate Program Duke University Durham North Carolina USA; ^2^ Medical Physics Graduate Program Duke Kunshan University Kunshan China; ^3^ Department of Radiation Oncology Duke University Medical Center Durham North Carolina USA

**Keywords:** AI, artificial intelligence, IMRT QA

## Abstract

We demonstrate a virtual pretreatment patient‐specific QA (PSQA) procedure that is capable of quantifying dosimetric effect on patient anatomy for both intensity modulated radiotherapy (IMRT) and volumetric modulated arc therapy (VMAT). A machine learning prediction model was developed to use linear accelerator parameters derived from the DICOM‐RT plan to predict delivery discrepancies at treatment delivery (defined as the difference between trajectory log file and DICOM‐RT) and was coupled with an independent Monte Carlo dose calculation algorithm for dosimetric analysis. Machine learning models for IMRT and VMAT were trained and validated using 120 IMRT and 206 VMAT fields of prior patients, with 80% assigned for iterative training and testing, and 20% for post‐training validation. Various prediction models were trained and validated, with the final models selected for clinical implementation being a boosted tree and bagged tree for IMRT and VMAT, respectively. After validation, these models were then applied clinically to predict the machine parameters at treatment delivery for 7 IMRT plans from various sites (61 fields) and 10 VMAT multi‐target intracranial radiosurgery plans (35 arcs) and compared to the dosimetric effect calculated directly from trajectory log files. Dose indices tracked for targets and organs at risk included dose received by 99%, 95%, and 1% of the volume, mean dose, percent of volume receiving 25%–100% of the prescription dose. The average coefficient of determination (*r* ^2^) when comparing intra‐field predicted and actual delivery error was 0.987 ± 0.012 for IMRT and 0.895 ± 0.095 for VMAT, whereas *r* ^2^ when comparing inter‐field predicted versus actual delivery error was 0.982 for IMRT and 0.989 for VMAT. Regarding dosimetric analysis, *r* ^2^ when comparing predicted versus actual dosimetric changes for all dose indices was 0.966 for IMRT and 0.907 for VMAT. Prediction models can be used to anticipate the dosimetric effect calculated from trajectory files and have potential as a “delivery‐free” pretreatment analysis to enhance PSQA.

## 1 INTRODUCTION

Pretreatment patient‐specific QA (PSQA) is an integral part of the intensity modulated radiotherapy (IMRT) and volumetric modulated arc therapy (VMAT) radiotherapy process and traditionally includes a plan specific verification measurement^1^, ^2^ with a comparative analysis using gamma index,^3^ as well as an independent dose calculation. Recommendations for the IMRT measurement‐based verification were recently provided by the AAPM Task Group 218, which included suggested tolerance limits and methodologies.^4^ Regarding the independent dose calculation, AAPM has long recommended that an independent check of MU calculation be performed within 48 h of the start of treatment,^5^, ^6^ with specific recommendations for non‐IMRT^7^ and IMRT^8^ plans being provided more recently.

Pretreatment PSQA measurement and analysis have known limitations, which, along with recent technological developments in radiotherapy, now warrant a reexamination of the PSQA process. Studies show that PSQA using gamma‐index analysis is a poor predictor of clinically relevant dosimetric errors^9^, ^10^ even after accounting for limitations from the measurement device itself,^11^ and the pretreatment measurement has been shown to be ineffective at identifying unacceptable plans.^12^ Emerging online adaptive radiotherapy (OART) techniques^13^, ^14^ are not compatible with a pretreatment PSQA measurement.^15^, ^16^ In addition, the independent dose calculation portion of PSQA has evolved from a simple calculation at a single point^6^, ^7^ to a volumetric calculation using convolution–superposition^17^, ^18^ or Monte Carlo,^19^, ^20^ which is now much more effective at identifying problematic treatment plans than is the pretreatment QA measurement.^21^ The acute need to revisit the PSQA paradigm is highlighted by the recent decision by the AAPM in 2022 to organize a special meeting on IMRT PSQA with the goal to reenvision how QA for IMRT fits into a comprehensive, safety‐focused operation.

One innovation in this regard is the application of artificial intelligence (AI) to enhance the PSQA process. Valdes et al. developed an algorithm to predict gamma‐index passing rates from IMRT PSQA using a weighted Poisson regression with Lasso regularization,^22^ which was later validated in a multi‐institutional study.^23^ Convolutional neural networks were also applied and compared in a separate study.^24^ Granville et al. incorporated treatment plan characteristics and routine linear accelerator quality control test results in the prediction algorithm.^25^ Other recent studies have included machine learning algorithms to predict the gamma‐index passing rates for IMRT PSQA using portal dosimetry,^26^ and VMAT PSQA using the ArcCHECK helical diode array,^27^, ^28^ the MatriXX ion chamber array,^29^ and the MapCHECK diode array.^30^ These studies demonstrate that AI can provide an accurate prediction of gamma‐index passing rates for IMRT and VMAT. Proposed clinical applications include using predicted passing rates to define plan‐specific passing thresholds,^22^, ^23^, ^24^ providing physicists advanced notice for cases that may fail PSQA to modify the plan or flag such cases for more rigorous evaluation,^25^, ^26^, ^27^, ^29^, ^30^ and replacement for PSQA delivery in selected instances.^24^, ^26^, ^29^

However, a remaining challenge^31^ is that an AI prediction model will still be subject to the same limitation of the original measurement: namely, being a poor predictor of clinically relevant dosimetric errors.^9^, ^10^ Two factors that contribute to this poor correlation include (1) limitations in the measurement being propagated into the comparative measure, and more importantly, (2) an inherent difference between dose agreement in phantom and dosimetric effect in the clinical plan. An example of the first of these is a limitation in the EPID fluence prediction algorithm causing discrepancies at the detector edge that, in turn, decreases the QA passing rate (due to false positives),^32^ which will then be inherently reflected in the prediction model.^22^, ^26^, ^31^ Regarding the second, although this limitation can be mitigated for a full pretreatment measurement by translating the measurement results back to the patient geometry (such as using fluence measured during QA to calculate dose on patient anatomy thus obtaining a “measured” patient DVH),^11^ a prediction model only provides the final comparative measure per plan or per field (as opposed to per detector); thus it does not provide sufficient information to reconstruct the dosimetric effect on patient anatomy.

For this study, we demonstrate an alternative virtual pretreatment PSQA strategy that circumvents these limitations by directly predicting the linear accelerator machine parameters at delivery (as opposed to predicting gamma passing rates from pretreatment measurement devices). Specifically, discrepancies in machine parameters that occur at treatment delivery for a new treatment plan are predicted using the planned linear accelerator parameters from the approved DICOM‐RT plan. The predicted plan parameters are then incorporated into a new DICOM‐RT plan with machine parameters modified to those “predicted” to occur at treatment delivery and dose is calculated on the patient geometry in conjunction with an independent dose calculation algorithm (Monte Carlo for this study) to directly determine the clinical dosimetric effect. Potential advantages of this technique include the following: (1) It is compatible with OART, (2) it provides direct feedback regarding the dosimetric effect on the patient anatomy, (3) the prediction can easily be validated for each patient after the first (and each subsequent) fraction by comparison with the trajectory file, and (4) it dovetails with existing trajectory file analysis and sophisticated independent dose calculation QA strategies.

## 2 METHODS AND MATERIALS

### 2.1 Overview

**FIGURE 1:**
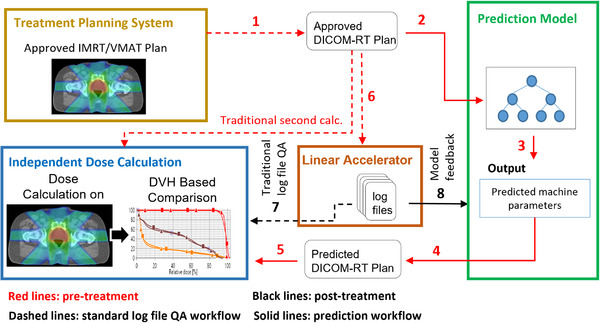
Workflow for virtual PSQA process in which a machine learning prediction of linear accelerator parameters is used to calculate dose on the patient anatomy prior to treatment delivery. Accuracy of the prediction model is then assessed after the first (and subsequent) fraction(s) using trajectory files. PSQA, patient‐specific QA

The proposed workflow for the virtual PSQA method is illustrated in Figure 1. After a new treatment plan is created and approved for clinical delivery, (1) the DICOM‐RT plan is exported from the treatment planning system and (2) imported into the prediction tool. Next, (3) a machine learning model that was previously trained using a dataset of approved DICOM‐RT plans and associated trajectory files from prior patients is used to predict discrepancies in machine parameters at delivery for the new treatment plan. Predicted machine parameters at treatment delivery are then calculated and incorporated into a new DICOM‐RT treatment plan (4) that is used to calculate doses on the patient geometry and perform a DVH‐based analysis (5) in conjunction with the Monte Carlo independent dose calculation. The robustness of the prediction model for the specific treatment plan can then be directly validated using the trajectory files that are passively acquired at the first and subsequent treatment fractions (7 and 8).

In this study, we demonstrate the feasibility of this technique by carrying out and validating a clinical implementation of the workflow in Figure 1. Specifically, a dataset consisting of DICOM‐RT plans and associated trajectory (log) files from previously treated patients was separated into a training set, and a testing set using an 80/20 split and independent prediction models were prepared for IMRT and VMAT. After training and validation of the model, the model was used to perform a clinical dosimetric analysis by predicting the dosimetry at treatment delivery for an altogether new set of patients and validated after treatment delivery by comparison to an analysis with the acquired trajectory files.

### 2.2 Prediction model

**FIGURE 2:**
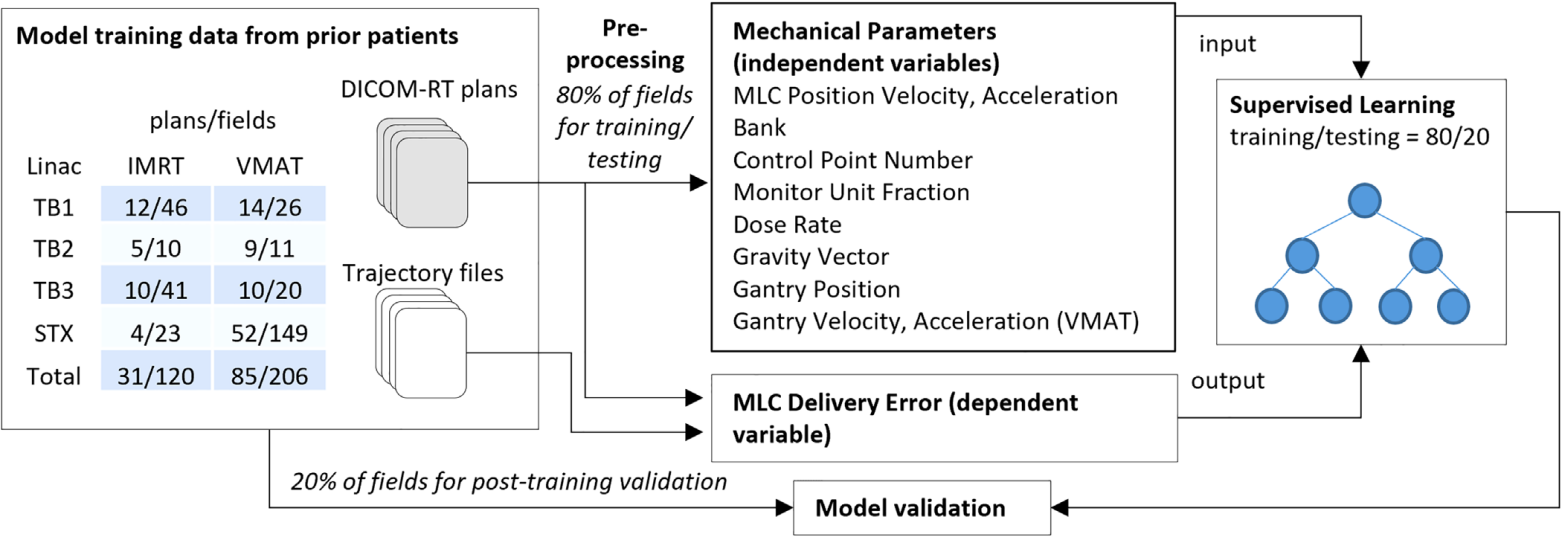
Data and methods for training and validation of machine learning model to predict machine parameters at treatment delivery from a new DICOM‐RT treatment plan

The data and methods for preparing the machine learning model to predict machine parameters at treatment delivery are summarized in Figure 2. Approved DICOM‐RT plans and associated trajectory files from prior patients were randomized into one set for training/testing (80% of fields) and one set for post‐training validation (20% of fields). The data then underwent preprocessing, in which for each control point the linear accelerator mechanical parameters were extracted, which serve as the input to the prediction model; as well as the difference between the actual and planned positions per MLC leaf, which serve as the desired output of the prediction model. The prediction model is applied to all MLCs indiscriminately, so that at each control point, each MLC leaf serves as a separate input sample. Thus, the number of input samples available for model training and validation from a single plan will be 120 MLCs multiplied by the number of control points. The prediction model was trained iteratively using an 80/20 data split for model training and testing, and separate models were created for IMRT and VMAT. After the model was finalized, it was evaluated using the 20% of data reserved for model validation. The resulting model from Figure 2 serves as the prediction model for the virtual PSQA workflow in Figure 1. In addition to the details provided later, further details regarding the preprocessing, interpolation, and training of the prediction model have been described previously.^33^, ^34^

#### 2.2.1 Training data from prior patients

Data for training the model consisted of trajectory files and DICOM‐RT plans for 120 unique IMRT fields (877, 098 control points and 105, 251, 760 input samples) and 206 unique VMAT fields (1208, 442 control points and 145, 013, 040 input samples) acquired from four separate linear accelerators between May 2019 and June 2019. Plans were acquired at Duke University Department of Radiation Oncology and prepared in the Eclipse treatment planning system v15.1 (Varian Medical Systems, Palo Alto, CA). Two models of linear accelerators were used to deliver the plans: TrueBeam STx (Varian Medical Systems, Palo Alto, CA) equipped with an HD120 MLC high‐definition multileaf collimator (Varian Medical Systems, Palo Alto, CA) and TrueBeam (Varian Medical Systems, Palo Alto, CA) equipped with Millennium 120 Leaf MLC (Varian Medical Systems, Palo Alto, CA). Our prior work demonstrated that a single combined prediction model can be created for these two models of accelerator.^34^

#### 2.2.2 Data preprocessing

Data preprocessing and model construction were primarily carried out using Python 3.7 (Python Software Foundation, Wilmington, DE). MATLAB R2019a (The MathWorks, Inc., Natick, MA) was also used for data validation and analysis. Linear accelerator mechanical parameters extracted at each control point that serve as inputs to the prediction model included the following: MLC position, MLC velocity, MLC acceleration, MLC bank, control point number, monitor unit fraction, dose rate, gantry angle, gravity vector^34^ (defined as the gravitational pull on each MLC, which is dependent on gantry angle), and in the case of VMAT, gantry velocity and gantry acceleration. Each control point serves as a separate input sample for model training and validation; thus the total number of features used in the model was 9 for IMRT and 11 for VMAT.

Features were normalized when necessary. As components accelerate to/from a static position at the beginning and ending of a treatment delivery, the control point number was normalized to be between 0 and 1. By normalizing control point number to a value between 0 and 1, all values falling near 0 will be near the beginning of the treatment delivery, and all values falling near 1 will be near the end of the treatment delivery. For the dose rate, the linear accelerator photon energies have different maximum dose rates (600 for 6X, 10X, and 15X, 1400 for 6XFFF, 2400 for 10XFFF). Thus, the instantaneous dose rate was normalized to the maximum dose rate, so as to avoid any differences in magnitude between photon energies.

The TrueBeam system records the linear accelerator machine parameters every 20 ms that are stored in a trajectory file in binary format, which were extracted using an in‐house Python 3.7 (Python Software Foundation, Wilmington, DE) script in conjunction with the log analyzer module in Pylinac.^35^ These values were then interpolated to the integer control point values found in the DICOM‐RT plan; an analysis of a subset of IMRT and VMAT fields showed the uncertainty introduced from this interpolation to be small with the difference between linear and cubic interpolation being <0.01 mm and <10% of the total error magnitude being predicted. The values from the DICOM‐RT were used as the “planned” machine parameters because, per a recent study, the majority of difference between planned and delivered machine parameters (>80%) is introduced during the conversion process at the linear accelerator from a DICOM‐RT plan to a series of deliverable machine parameters, rather than at the treatment delivery itself.^33^ Thus actual versus expected positions in the trajectory file is only ∼20% of the discrepancy in machine parameters at delivery; in order to capture the total delivery error, any trajectory file analysis must compare the trajectory file directly to the DICOM‐RT file.^33^

When the machine parameters were calculated from the DICOM‐RT file rather than from the trajectory file (during model validation in Figure 2 and QA workflow in Figure 1), some parameters used for the prediction model are not explicitly defined within the DICOM‐RT file (such as dose rate, MLC velocity, MLC acceleration, gantry velocity, and gantry acceleration) but were instead calculated accounting for mechanical limitations of the linear accelerator. The output from the prediction model is the discrepancy per MLC leaf between the recorded position at treatment delivery (actual) recorded by the trajectory file and the original position recorded in the DICOM‐RT plan (planned). When training the model, this value was calculated and extracted from the DICOM‐RT and trajectory files. When the prediction model is used in the PSQA workflow in Figure 1, its output provides the value to be added to the original DICOM‐RT planned position to arrive at the predicted “actual” values at treatment delivery.

#### 2.2.3 Model selection and supervised training

Prediction models were built using the Scikit‐learn toolkit.^36^ The target response of these models was set to the MLC delivery error, and the feature variables of the models were set to the mechanical parameters described previously. During each iteration of the model training process, the training data was randomly split into training (80%) and testing (20%) subgroups. A number of prediction models were evaluated, including linear regression,^37^ decision trees,^38^ ensemble algorithms (e.g., boosting, bagging),^39^ and neural networks.^40^ The final model(s) utilized one of two advanced decision‐tree models, a boosted tree and a bagged tree. Both models begin with separating the input data into multiple subsets. The boosted‐tree model randomly selects and trains a subset to create a decision tree, and the other subsets are trained sequentially using the previously trained decision tree. In contrast, the bagged tree independently trains each subset into a decision tree, with the result being the average of all predictions from different trees.^39^

#### 2.2.4 Model validation

After the final prediction models were selected and trained, their accuracy at predicting machine parameters at treatment delivery was quantified for the 20% of plans that were allocated for validating the model, which had not been utilized in training the model (see Figure 2). Model accuracy was assessed by comparing predicted MLC positions (predicted MLC discrepancy + planned MLC position) to the actual MLC positions determined from the trajectory file relative to the DICOM‐RT file.

### 2.3 Clinical implementation

After the prediction model was selected, trained, and validated, it was implemented clinically following the workflow in Figure 1. Note that the model validation dataset was completely separate from the clinical implementation dataset. For the independent dose calculation, a Monte Carlo–based dose calculation was employed, which is the same software and dose calculation used clinically for a pretreatment‐independent volumetric dose calculation^19^ (SciMoCa Version 1.5.1.2890, Scientific‐RT, Munich Germany). The Monte Carlo dose engine shares fundamental concepts with the voxel Monte Carlo (VMC) family of codes (such as VMC++ and XVMC) along with a virtual source model. Commissioning of the dose engine for clinical use was carried out previously, which included the use of independent, institution‐specific beam data.

#### 2.3.1 Dosimetric analysis

The clinical workflow was carried out for 7 IMRT treatment plans (breast x1, lung SBRT x1, head and neck x3, prostate and lymph nodes x1, gynecological pelvis and lymph nodes x1) with a total of 61 IMRT fields, as well as 10 VMAT plans (single isocenter multi‐target radiosurgery x10) with a total of 35 VMAT arcs. The Monte Carlo–independent dose calculation was carried out on the original DICOM‐RT plan using the machine parameters derived from the prediction model (steps 1–5 from Figure 1), as well as the machine parameters recorded from the trajectory file at treatment delivery (step 7 from Figure 1). Dosimetric statistics were tabulated for all gross tumor volumes, clinical tumor volumes, and planning target volumes (PTVs) and for selecting organs at risk (OARs). Dose statistics included the dose received by 99%, 95%, and 1% of the volume (*D*_99%_, *D*_95%_, and *D*_1%_), as well as the mean dose (*D*_mean_) and the percent of volume receiving the prescription dose (*V*_100%_). In order to facilitate the combined analysis of the IMRT plans from a variety of treatment sites, a single set of ring structures was used for the OARs. These included ring structures located 0–3 mm from the PTV edge (Ring_0‐_3 mm), 3–6 mm from the PTV edge (Ring_3‐_6 mm), and 6–9 mm from the PTV edge (Ring_6‐_9 mm). Dose statistics recorded for the ring structures were the same as those for the PTVs. The OAR used for the radiosurgery VMAT cases was the brain, with tabulated dose statistics for the percent of the brain receiving 12 Gy (*V*_12 Gy_), and the percentage of the brain receiving 20%, 50%, 75%, and 100% of the prescription dose (*V*_20%_, *V*_50%_, *V*_75%_, and *V*_100%_).

## 3 RESULTS

### 3.1 Model validation

**FIGURE 3:**
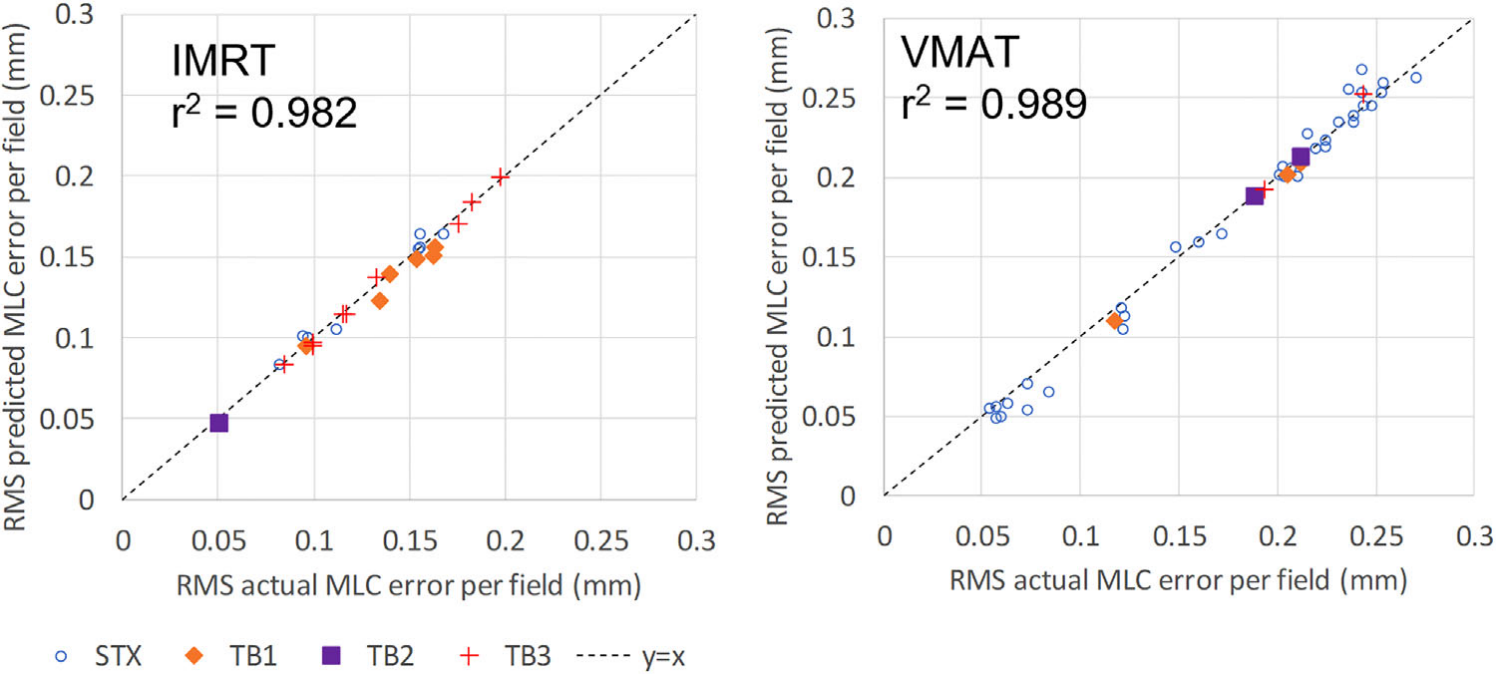
Prediction model performance for IMRT and VMAT for validation dataset. Each point represents a single field (*n* = 24 for IMRT, 41 for VMAT). RMS, root mean square

With the exception of the linear regression model, all prediction models performed similarly with mean absolute error (MAE) of 0.004–0.010 mm for IMRT and 0.021–0.034 mm for VMAT. The final selected prediction model was a boosted tree for IMRT and a bagged tree for VMAT based upon their performance. For the IMRT validation dataset (24 IMRT fields), the MAE was 0.008 mm compared to the MAE of the total difference between actual and planned MLC positions of 0.13 mm; thus the prediction model was accounted for 93.8% of the difference between planned and actual MLC positions for IMRT. For the VMAT validation dataset (41 VMAT fields), the MAE was 0.026 mm compared an MAE of 0.179 mm for the total MLC error; thus the model was accounted for 85.5% of the difference between planned and actual MLC positions for VMAT. The performance of the models for the validation dataset is further illustrated in Figure 3. For IMRT, the average coefficient of determination (*r* ^2^) for each individual field when comparing the predicted and recorded delivery errors for each control point was 0.987 ± 0.012 [0.953, 0.997]. For the VMAT model, this value was 0.895 ± 0.095 [0.453, 0.964]. When comparing the root mean square (RMS) of the MLC delivery error per field, *r* ^2^ of the predicted versus actual RMS values was 0.982 for IMRT and 0.989 for VMAT.

### 3.2 Clinical implementation: Dosimetric analysis

**FIGURE 4:**
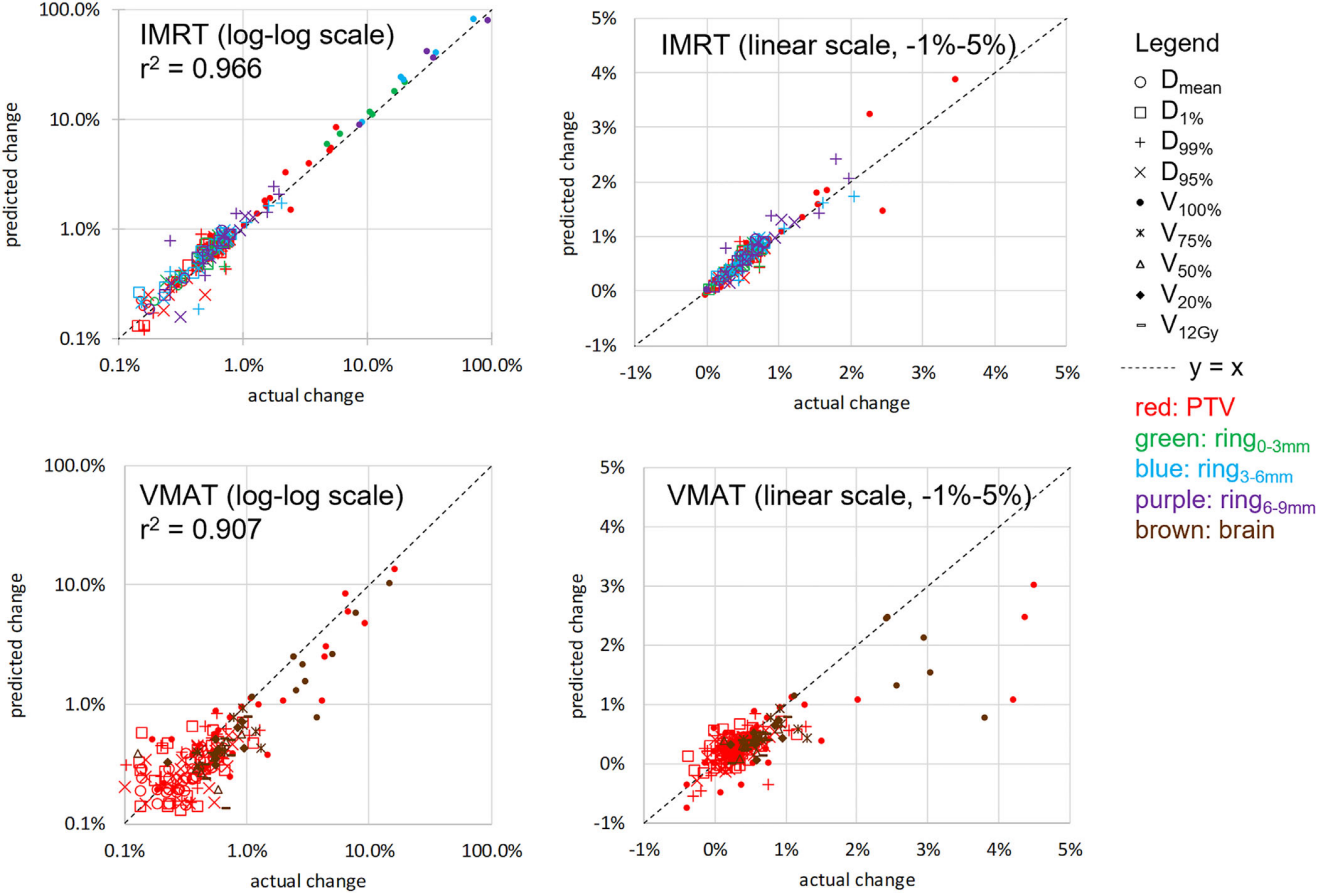
Change in dosimetric indices after accounting for machine parameters at treatment delivery for the 7 IMRT and 10 VMAT cases used for the dosimetric analysis. Dosimetric effects predicted pretreatment by the prediction model are highly correlated (*r* ^2^ > 0.9) with the effect derived using the posttreatment trajectory file

**FIGURE 5:**
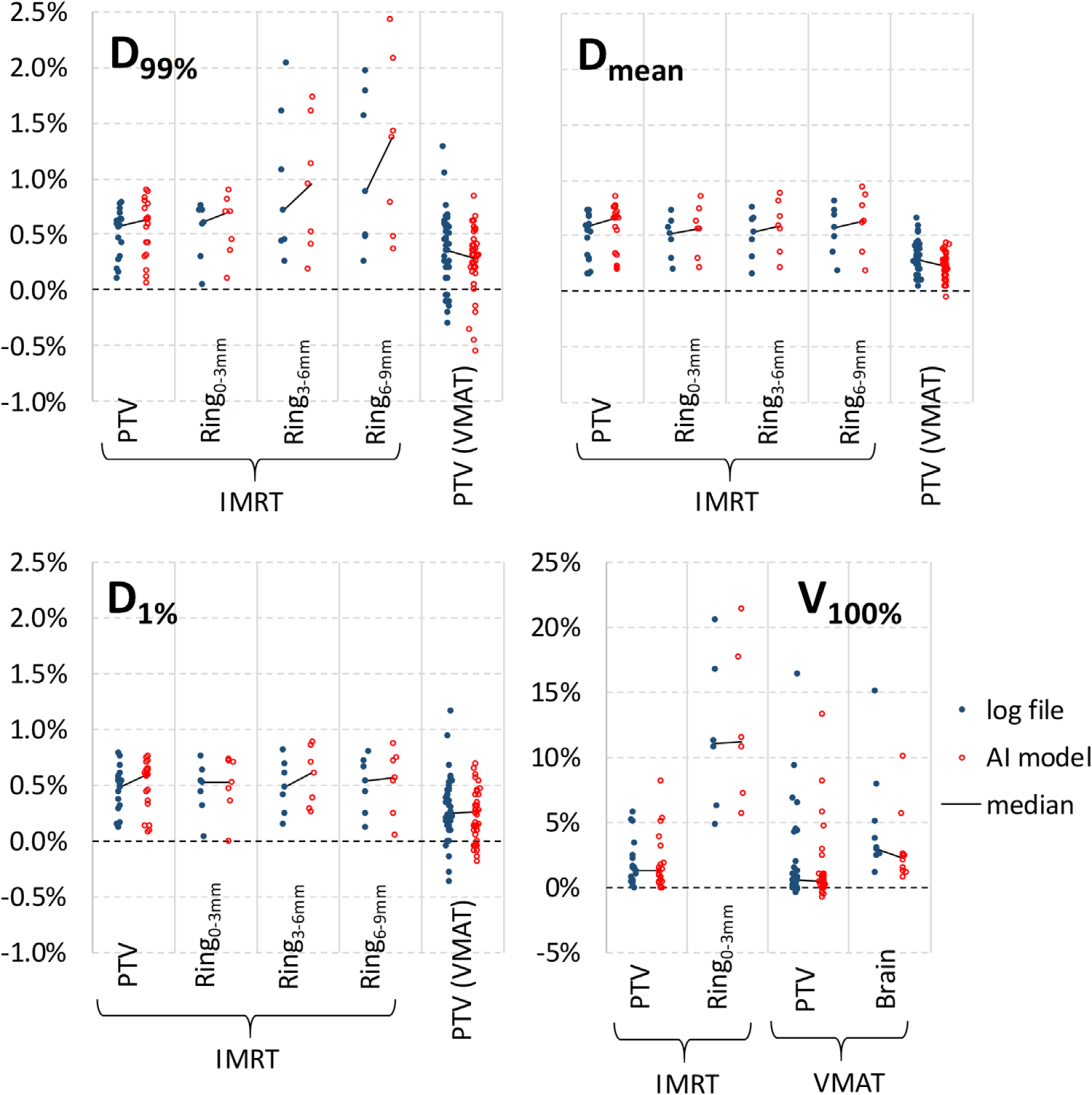
Change per dosimetric index after accounting for machine parameters at treatment delivery for 7 IMRT and 10 VMAT cases used for dosimetric analysis

The virtual PSQA procedure from Figure 1 was carried out for the 7 IMRT and 10 VMAT plans. Dose for all plans (original, trajectory file, and prediction) was recalculated with the Monte Carlo–independent dose calculation software using the patient's original CT and structure set. Figure 4 shows the correlation of predicted and actual changes in dosimetric indices due to discrepancies in machine parameters at delivery (*r* ^2^ = 0.966 for IMRT, *r* ^2^ = 0.907 for VMAT). For this set of patients and set of dosimetric indices, the sensitivity (true positive rate) and specificity (true negative rate) of detecting a 5% change in any of the dosimetric indices on the patient anatomy was 100% and 99.4%, respectively, for IMRT, and 71.4% and 100%, respectively, for VMAT. In Figure 5, the magnitude of dosimetric effects observed (from trajectory files and predicted by model) is further visualized via stratification by dose index. From this figure, it is clear that most dosimetric effects are modest: changes in the near minimum dose (*D*_99%_), mean dose (*D*_mean_), and near maximum dose (*D*_1%_) are typically within ±1%. One exception is that slightly larger dose effects are observed for *D*_99%_ of the Ring_3‐_6‐mm and Ring_6‐_9‐mm structures. There was also notable change in percent of volume receiving the prescription dose (*V*_100%_) both for the PTV and adjacent OARs; in a number of cases, the change in *V*_100%_ was in excess of 5% and 10%.

## 4 DISCUSSION

In this study, we applied a machine learning model to predict linear accelerator machine parameters at treatment delivery that could then be used to calculate the effect of discrepancies on the patient DVH, and we proposed a framework to utilize this prediction model to enhance PSQA. Various prior studies have utilized trajectory files for PSQA measurement or evaluated correlation of recorded errors with various plan characteristics.^41^, ^42^, ^43^, ^44^, ^45^ In addition to these studies, a few prior studies demonstrated that machine parameters at delivery can be predicted using machine learning models trained using trajectory files from previous patients. Carlson et al. used DICOM‐RT and MLC Clinac Dynalog files from 74 VMAT plans to train and validate a machine learning model to predict machine parameters.^46^ Osman et al. trained a similar prediction model using the Clinac Dynalog files for 10 sliding window IMRT plans.^47^ We also recently released open‐source models for both IMRT and VMAT in a prior study trained using the TrueBeam trajectory files from 142 IMRT and 125 VMAT plans taken from nine separate linear accelerators from two institutions^34^; however, one limitation to the prior work is that it accounted for only ∼20% of the delivery error as it did not include conversion error.^33^ These three prior studies concur with the results from the current study in demonstrating the effectiveness of machine learning models trained using prior trajectory files to predict linear accelerator machine parameters at treatment delivery for a new treatment plan.

Recently, a number of virtual PSQA techniques have been proposed that utilize an AI model to predict measurement‐based PSQA results such as gamma index or dose difference.^22^, ^23^, ^24^, ^25^, ^26^, ^27^, ^28^, ^29^, ^30^ This study also involves a virtual PSQA technique that utilizes a predictive model; however, instead of predicting gamma index, the dosimetric effect on the patient anatomy is determined directly using a prediction model based on linear accelerator trajectory files. This has the advantage of overcoming the poor correlation of gamma index with clinically relevant dosimetric errors.^9^, ^10^ A second advantage is the preponderance of data for training the model: When predicting gamma index, each manual QA delivery and analysis provides a single data point for training, whereas linear accelerator trajectory files are recorded passively, and each delivery includes hundreds to thousands of data points for training (number of control points × 120). Thus, with trajectory files, it is possible to continuously update the model on a regular, even daily, basis as new trajectory files are recorded. However, one major disadvantage of the technique proposed here compared to other virtual QA techniques that have been proposed is that the training data for the model utilized trajectory files rather than independent QA measurements. Trajectory files include the machine parameters recorded by the linear accelerator itself, which in some instances may not reflect the true position, due to calibration errors or failing MLC components.^48^ Beam characteristics such as beam symmetry and variations in output per monitor unit are also not reflected in the trajectory file. Because of this, current PSQA strategies involving trajectory files are commonly coupled with increased frequency of MLC QA. Another possibility is to incorporate more information into the prediction model from routine machine QA; for instance, fluctuations in output, beam symmetry, MLC, couch, collimator, and gantry accuracy are already measured during various routine QA tests (such as daily QA, monthly QA, and automated machine performance checks), and this information could be incorporated into the PSQA prediction model. This could thus enable a PSQA process that is virtual (prediction made immediately after treatment planning using prior trajectory files), patient specific (prediction made using patient DICOM plan), based on measurement (by incorporating information from routine QA), and includes DVH‐based analysis. This possibility is an area of focus for future work on this project.

In this study, the ultimate outcome determined from prediction model (and independent dose calculation) is the dosimetric effect on the patient anatomy. One question that was not addressed in this work is defining action criteria for DVH metrics from PSQA. The AAPM Task Group 219 provides some guidance for acceptable dose differences to be observed for independent dose calculations^8^; however, these recommendations technically apply to point dose values rather than volumes. Ultimately, the best patient‐specific one may be defined based on absolute dose constraints from QUANTEC, etc. especially for special cases (retreatment, nearby critical OARs, etc.).

One ongoing shift in the paradigm of PSQA is the importance of the independent dose calculation, as it has been shown to be much more effective at identifying unacceptable treatment plans than is the PSQA measurement.^21^ In addition, for a trajectory file approach, aspects of the treatment plan that have been traditionally verified by measurement QA, but are not verified using a trajectory file approach (such as appropriateness of the beam modeling for the patient specific plan), are instead verified via the more sophisticated Monte Carlo–independent dose calculation (as opposed to a simple hand calculation). In this study, we utilized the Monte Carlo–independent dose calculation algorithm when quantifying the dosimetric effect on patient anatomy from the prediction/trajectory files. An equally valid approach would have been used for the primary treatment planning system when recalculating dose from the prediction model and trajectory file(s), while still carrying out a separate independent dose calculation using the original treatment plan. It should be noted that the dosimetric analysis (such as in Figure 4) utilized the Monte Carlo–independent dose calculation algorithm for both the predicted and actual doses, so that the analysis does not include dosimetric effects due to differences between the primary and secondary dose calculation algorithms.

Although Monte Carlo is considered a “gold standard” for dose calculation, it should be noted that its accuracy is dependent upon its implementation, and it can be subject to similar limitations of other dose calculation algorithms.^8^ With these limitations in mind, the independent dose calculation is best viewed as a “second opinion” rather than a “gold standard” and should be commissioned following published recommendations, ideally using a fundamentally different dose algorithm tuned to match independent beam data rather than matching the primary algorithm.^7^, ^8^ Regarding these limitations, one possible mitigation strategy (which may be explored in future work) is to expand the independent dose calculation to include a plan robustness check, in which dose is recalculated multiple times while introducing perturbations in the beam model, the magnitude of which represents realistic uncertainty in the model attributes.

More broadly, the question of effectiveness of a prediction model for trajectory file PSQA, and how to best incorporate it into a PSQA process, would be best evaluated using a failure modes and effects analysis (FMEA).^49^ Potential questions that could be addressed by an FMEA analysis include (1) What value is added in incorporating the prediction model PSQA process with the current PSQA process? (2) What changes to the current PSQA process are justified when the prediction model is incorporated (such as performing trajectory file QA using fraction 1 rather than from a pretreatment delivery)? (3) Is a virtual QA process a sufficient replacement for the physical QA (and in which circumstances), and what other QA tests (increased frequency of routine QA, enhanced MLC QA, commercially available plan checks, plan robustness analysis, etc.) may be required to mitigate added risks in this situation? Such an analysis is planned for future work.

## 5 CONCLUSION

In this study, we demonstrated the feasibility of a virtual PSQA in which the machine parameters at treatment delivery are predicted directly using a machine learning model trained using prior trajectory files, and for which the dosimetric effect on the patient anatomy is calculated directly via a Monte Carlo–independent dose calculation. The predicted and actual RMS error in machine parameters per field agreed with *r* ^2^ of 0.982 for IMRT and 0.989 for VMAT. The dosimetric parameters on the patient anatomy were predicted with *r* ^2^ of 0.966 for IMRT and 0.907 for VMAT. Machine learning models can predict ∼90+ percent of the dosimetric effect calculated from trajectory files and have potential as a “delivery‐free” pretreatment analysis to enhance PSQA. Future work will include FMEA to determine how to best incorporate this tool into a clinical workflow.

## AUTHOR CONTRIBUTION

Lam M. Lay contributed to the programming, experiments, analysis, and writing. Kai‐Cheng Chuang assisted in the programming and analysis. Yuyao Wu assisted in the programming and analysis. Dr. William Giles contributed to the research study design and analysis. Dr. Justus Adamson contributed to the research study design, analysis, and writing.
